# An Unusual TEN-Like Presentation of Juvenile Bullous Pemphigoid: A Diagnostic Challenge

**DOI:** 10.1155/2022/8507156

**Published:** 2022-04-11

**Authors:** Zahra Nikyar, Parvaneh Hatami, Zeinab Aryanian, Soheila Sotoudeh, Vahid Ziaee, Azadeh Goodarzi

**Affiliations:** ^1^Autoimmune Bullous Diseases Research Center, Tehran University of Medical Sciences, Tehran, Iran; ^2^Department of Dermatology, Skin Diseases and Leishmaniasis Research Center, School of Medicine, Isfahan University of Medical Sciences, Isfahan, Iran; ^3^Department of Dermatology, Babol University of Medical Sciences, Babol, Iran; ^4^Department of Dermatology, Children's Medical Center, Center of Excellence, Tehran University of Medical Sciences, Tehran, Iran; ^5^Pediatric Rheumatology Research Goroup, Rheumatology Research Center, Tehran University of Medical Sciences, Tehran, Iran; ^6^Department of Pediatrics, Tehran University of Medical Sciences, Tehran, Iran; ^7^Department of Dermatology, Rasool Akram Medical Complex Clinical Research Development Center, School of Medicine, Iran University of Medical Sciences, Tehran, Iran; ^8^Skin and Stem Cell Research Center, Tehran University of Medical Sciences, Tehran, Iran

## Abstract

Bullous pemphigoid (BP) is an acquired autoimmune bullous disorder rarely seen in the pediatric population. It usually presents as large and tense bullae, predominantly distributed in the acral areas. Herein, we describe a case of childhood BP with an atypical presentation mimicking toxic epidermal necrolysis (TEN). This case shows us that juvenile BP should be considered in the differential diagnosis of TEN in children, particularly if there are unusual features and an intractable course.

## 1. Introduction

Bullous pemphigoid (BP) is an acquired autoimmune bullous disorder rarely seen in the pediatric population [1]. BP is characterized by linear deposition of autoantibodies and C3 along the dermoepidermal junction (DEJ), leading to a subepidermal bullae formation. It usually presents as large and tense bullae with a predominant acral involvement [2]. The exact etiopathogenesis of BP is not fully understood, but some infections, medications, and vaccines, along with genetic susceptibility, have been shown to play a role [3–6].

Here, we present a 3-year-old patient with an atypical presentation of BP whose clinical features were perplexing and provided a distinct diagnostic challenge.

## 2. Case Presentation

A 3-year-old girl presented to a general clinic with generalized vesiculobullous lesions. Her parents reported that symptoms began as pruritic, red, and scaly lesions on her feet which were initially diagnosed as eczema and treated with emollients and topical steroids. However, despite the treatment, the lesions progressed and extended to the abdomen and upper extremities within one month. Extension of the lesions raised suspicion of a scabies infestation. Permethrin cream 5% was prescribed which was not effective. Parents also reported the use of an unknown herbal remedy for a week which led to the appearance of some new vesiculobullous and erosive lesions.

**Figure 1:**
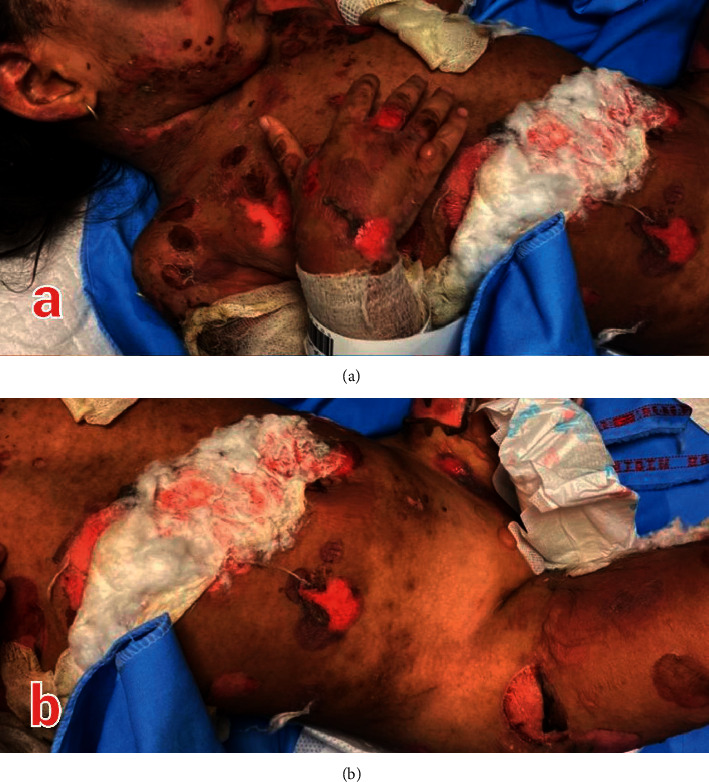
Generalized erosions and flaccid bullae (a, b).

The patient was referred to our hospital, Markaz e Tebi Atfal, Tehran University of Medical Sciences (TUMS), Tehran, Iran, at February 2021, when she was ill with generalized erythematous patches, plaques, and flaccid bullae filled with clear fluid, along with some erosions, distributed all over her body (Figure 1). Nikolsky's sign was positive.

Mucosal examination revealed erosive lesions in the buccal area, with minor erythema on the vaginal mucosa; the ocular mucosa was normal.

No positive family history of any dermatosis was noted.

Lab tests: WBC, 17900 (cell/mcL); lymph, 4300 (cell/mcL); poly, 12800 (cell/mcL); mono, 1350 (cell/mcL); Hb, 14 (g/dL); RBC, 5.38 (millions); MCV, 77.9 (fL); Plt, 686000 (cell/mcL); FANA, negative; CRP, 67 (mg/L); ESR, 9 (mm/h); IgG,1466 (mg/dL); IgM, 29 (mg/dL); IgA, 54 (mg/dL). Smear and cultures taken from intact bullae were negative. Blood culture was positive for *S. aureus*.

Initially, based on the extensive bullous and erosive lesions, positive Nikolsky's sign, and mucosal involvement, it was assumed to be a case of toxic epidermal necrolysis (TEN) possibly caused by the unknown herbal remedies. However, several points did not support this diagnosis; there was no history of any systemic medication; the course of presentation was subacute; erythema in the vaginal mucosa was considered to be normal by the pediatric dermatologist; and oral lesions were less severe than normally seen in TEN. Other differential diagnoses considered were staphylococcal scalded skin syndrome (SSSS), pemphigus vulgaris, bullous pemphigoid, and bullous impetigo.

A 4 mm punch biopsy was taken from one of the new lesions for a histopathologic assessment and another from a perilesional skin for a direct immunofluorescence (DIF) evaluation.

**Figure 2:**
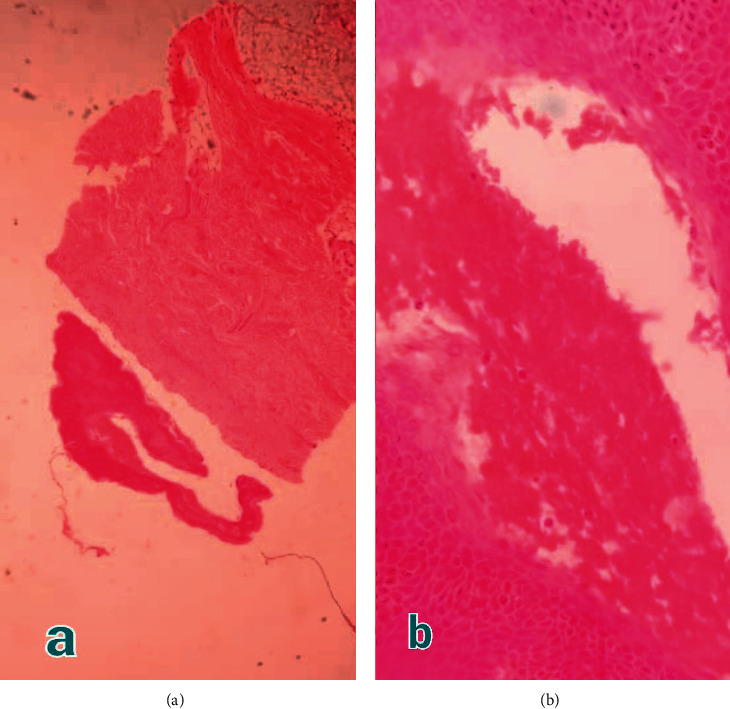
Distinct subepidermal bulla (H&E, x40) (a). Dermal infiltration of lymphocytes with numerous eosinophils compatible with the diagnosis of BP (H&E, x100) (b).

Histopathology showed distinct subepidermal bulla and dermal infiltration of lymphocytes with numerous eosinophils compatible with the diagnosis of BP (Figure 2).

**Figure 3:**
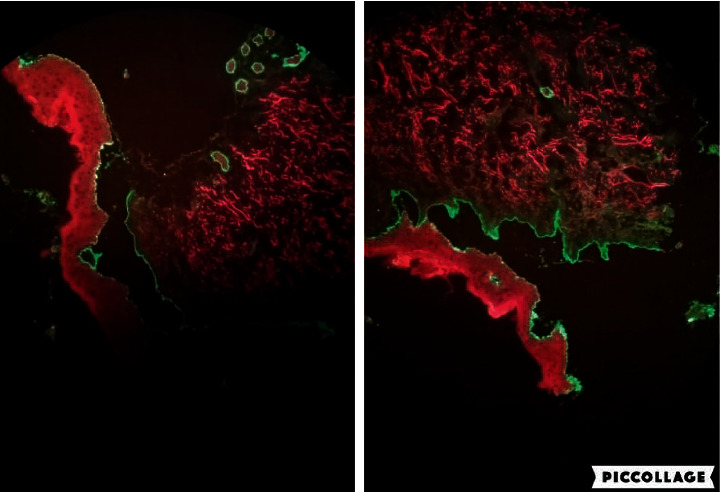
Linear deposition of IgG and C3 at DEJ.

DIF also revealed a linear pattern of C3 and IgG deposition at DEJ and confirmed the diagnosis of BP (Figure 3).

Initially, treatment had been started with IVIG (1 g/kg/day for three days) due to the involvement of >90% of the surface area of the body. However, the lesions were resistant to 3 cycles of IVIG, and new bullous lesions continued to appear; therefore, cyclosporine (3 mg/kg/day) was added to the treatment. After the diagnosis of BP was approved, treatment was continued with methylprednisolone pulse (30 mg/kg/day for three days), and the lesions went into remission after a few days. The treatment was continued on prednisolone (2 mg/kg/day) and dapsone 25 mg/day, and cyclosporine was discontinued. Eventually, prednisolone was tapered gradually within 3 months. At present, she is taking only 25 mg/day dapsone, and there has been no recurrence of lesions within the 5-month period of follow-up.

The patient's parents provided written informed consent for the publication of this case report and accompanying images.

## 3. Discussion

Bullous pemphigoid is a blistering disorder that mainly affects elderly patients and is rarely seen in the pediatric population [1]. The incidence of BP in the pediatric population is unknown [1].

The clinical presentation of pediatric BP differs from adults in terms of prevalent sites of involvement: palmoplantar and facial lesions are frequently seen in children, whereas flexural regions are most frequently involved in adults [2, 3].

Mucosal lesions are rare in BP, and if present, may be a sign of a more severe and refractory disease [7]. The presence of oral lesions in our case and the relatively intractable course of the disease further supports this idea. An unusual feature of this case was the presence of flaccid bullae and erosions rather than tense bullae, as well as mucosal involvement, which could mislead the clinician.

Moreover, lack of the positive history of any triggering factor, including infection, medication, or recent vaccination, along with the severe and intractable course of the disorder, made it even more challenging to diagnose before histopathologic evaluation. However, the final diagnosis in our case was made based on histopathologic and DIF data, and an indirect immunofluorescence (IIF) study was not performed for further confirmation of the diagnosis which should be considered as a limitation of this report.

TEN-like presentation of BP has been rarely reported in adults [8], but to the best of our knowledge, no similar article has been published regarding this issue in pediatric population.

Initial BP treatment is potent topical steroid ointments and systemic prednisolone [9]. According to a systematic review on 81 infants suffering from BP, prednisolone and dapsone were the most common monotherapies in pediatric BP [4].

However, due to the severity of the condition in our patient and the initial diagnosis of TEN, we had to consider more potent options, including IVIG (1 mg/kg/day for three days), cyclosporine (3 mg/kg/day), and methylprednisolone pulse to control the disease. After initial remission, dapsone (2 mg/kg) was added as a steroid-sparing agent, but it was reduced to 0.5 mg/kg due to the development of hemolytic anemia. Dapsone was well-tolerated by the patient, so prednisolone could be tapered successfully within 3 months. This result corresponds with a recent report of a refractory case of childhood BP that was successfully controlled by dapsone and systemic steroids [10].

## 4. Conclusion

We reported a case of childhood BP with an unusual presentation that clinically mimicked TEN to emphasize the necessity of paying more attention to history taking, physical examination, and clinical suspicion as key factors to reaching the correct diagnosis. Furthermore, one can infer from our report the priority of prescribing methylprednisolone pulse over IVIG and cyclosporine in hard-to-treat BP in children. However, further studies are needed to shed more light on this issue.

## Data Availability

The data used to support this study are available from the corresponding author upon request.
